# Comparative assessment of nutritional and functional properties of different sorghum genotypes for ensuring nutritional security in dryland agro-ecosystem

**DOI:** 10.3389/fnut.2022.1048789

**Published:** 2022-11-09

**Authors:** Maw Ni Soe Htet, Baili Feng, Honglu Wang, Lixin Tian, Vivek Yadav

**Affiliations:** ^1^State Key Laboratory of Crop Stress Biology for Arid Areas, College of Agronomy, Northwest A&F University, Xianyang, China; ^2^State Key Laboratory of Crop Cultivation and Farming System in Northwestern Loess Plateau, College of Agronomy, Northwest A&F University, Xianyang, China; ^3^Rice Bio Park Research Section, Post-Harvest Technology and Food Science Research Division, Department of Agricultural Research, Naypyidaw, Myanmar; ^4^State Key Laboratory of Crop Stress Biology in Arid Areas, College of Horticulture, Northwest A&F University, Xianyang, China

**Keywords:** sorghum varieties, nutritive values, quality traits, micrometric properties, functional properties

## Abstract

The cultivation of unique sorghum (resistant to abiotic stresses and re-recognized as healthy food) has attracted interest as an environmentally friendly minor cereal and may be a solution to food and nutritional security. However, information about how the use of selected sorghum grains affects nutritive values and its functional properties from sorghum flours is still lacking. To address this question, we selected six sorghum varieties (i.e., JinZa 34, LiaoZa 19, JinNuo 3, JiZa 127, JiNiang 2, and JiaXian) for the comprehensive analysis of the relationship among nutritional compositions, energy value contributions, and functional properties of sorghum grains. Results showed that Carr’s index (CI) and angle of repose (AR) of all sorghum flours indicated good flow and compressibility properties in terms of micrometric parameters. All sorghums were considered free of tannin. Based on the scatterplot analysis, the proportions of energy contributions due to protein, fat, and carbohydrate (CHO), were highly positively correlated with protein, fat, and CHO, respectively. The significantly different flours of six sorghum varieties resulted in different functional properties. The amylose content showed a highly negative association with light transmittance and water and oil absorption capacities. In addition, amylose had a highly positive relationship with water solubility (WS) and swelling power (SP). JinNuo 3 had the highest nutritional compositions [proximate, mineral, anti-nutritional values, and amino acid (AA) profiles] and functional properties indicating that it could be used as a brewing liquor. Our findings will provide a new opportunity to cultivate sorghum as an environment friendly minor cereal crop in dryland agro-ecosystems of arid and semi-arid regions of northern China for nutritional security, agriculture processing, and non-food industry in the future.

## Introduction

Sorghum [*Sorghum bicolor* (L.) Moench], an environmentally friendly crop, is resistance to water and fertilizer efficient than other major cereal crops, such as maize, wheat, and rice (1). Sorghum is a multipurpose minor cereal crop, primarily used as food, feed, and forage, and as important raw materials for brewing liquor (2) and value-added food products (3). According to the public health point of view, sorghum provides many health benefits due to its antioxidant, anti-inflammatory, anti-proliferative, anti-diabetic, and anti-atherogenic properties (4). Its phenolic compounds can prevent many diseases including cancer, diabetes, digestive tract disease, and cardiovascular disease (5, 6).

Sorghum is widely grown in dryland agricultural systems in arid and semiarid specific zones of northern and northeastern parts of China. In those areas, the excessive consumption of water and over-application of inorganic fertilizers have led to serious action on environmental problems for sustainable agriculture (7). These characteristics limit the productivity and make dryland agro-ecosystems both inherently dynamic and vulnerable. It is clear that small-scale farming will continue to play an important role in providing livelihood security for people in those areas. To reduce the poverty and malnutrition it is therefore necessary to improve the productivity of current farming systems, and at the same time safeguard the generation of other ecosystem services, on which local people also depend (7, 8). Therefore, sorghum crop, which is an environmentally friendly minor cereal crop and recognized as healthy food, may be a solution. Nowadays, sorghum research is crucial for environmentally friendly crop and most promising candidate crop as a sorghum-based intercropping for agricultural sustainability (8, 9). Intercropping and sorghum ratooning are popular cultivation techniques among small-scale farmers around the globe (3, 10, 11). Ratooning is a system that grows shortly after cutting the main crop, and the second crop is harvested in the same cropping season. This method is a feasible harvesting practice (11, 12). Sorghum intercropping with some legumes has many economic returns (13–15), and the sorghum ratooning practice also has several advantages, such as no need for new seed and land preparation, covering of time of sowing, and high grain yield and quality (12). Zhou et al. (16) reported that sorghum ratoon crop has high contents of starch, protein, and tannin and low fat content after application of nitrogen fertilizer. This chemical composition indicates good quality for liquor production.

The cultivation of sorghum has decreased many years ago, but has attracted interest recently because of its highly nutritive grains, bioactive compounds, and starch versality (4, 17, 18). Many cereal scientists and food processors extensively studied the characteristics and physicochemical properties of sorghum starches (amylose and amylopectin), which can be adapted for agricultural processing and non-food industry (19–21). The literature reported that the contents of amylose in sorghum flour and starch affect the functional and physicochemical properties (22, 23). Yang et al. (24) reported that flours and starches of non-waxy proso and foxtail millets had higher amylose contents than those of waxy proso and foxtail millets. Potato flours and starches, which have higher amylose content that affects pasting properties than sweet potato flours and starches, have wider applications as functional flours with high nutritive values (25). Some studies also reported that in Tartary buckwheat, the four has the higher amylose content and other nutritional compositions than the starch, thus indicating its suitability for application in the food processing industry (26). However, limited information is available on nutritional compositions, quality traits, physical and functional properties, and anti-nutritional traits in industrial usage of sorghum flours.

The consumption and production of sorghum crops first need vigorous assessment of the ability and feasibility of particular cultivar to provide suitable yield, quality and nutritional value. Moreover, investigation of perceived functional properties is essential to assess the consequences of industrial processing on the crops like mechanical or thermal treatment. This could be due to insolubilization of denatured protein isolates by industrial treatment which may have either beneficial or deleterious impact on the nutritive values of the crops (17, 20, 21). Hence in the present study, sorghum seed flours, obtained from six Chinese sorghum cultivars i.e., JinZa 34, LiaoZa 19, JinNuo 3, JiZa 127, JiNiang 2, and JiaXian have been assayed with a view to evaluating their nutritional and functional properties. After purifying and milling the sorghum seeds, the flours obtained were evaluated for micrometric properties, proximate composition, energy values and contribution, mineral and amino acid (AA) composition, and anti-nutritional traits. Among different functional properties, SP, WS, WAC, OAC, light transmittance, and LGC of the sorghum flours have been assayed. In short, the present study was designed in with the hope of establishing the significance of sorghum as a major cereal grain crop in the human diet and finding the most beneficial cultivar in terms of nutritional value and industrial applicability. Moreover, our findings will provide a new opportunity to cultivate sorghum in dryland agro-ecosystems in arid and semi-arid regions of northern China and the application of sorghum flours in meeting the demand of consumer preference in the food industry.

## Materials and methods

### Materials

Six sorghum varieties JinZa 34 (JZ 34), LiaoZa 19 (LZ 19), JinNuo 3 (JN 3), JiZa 127 (JZ 127), JiNiang 2 (JN 2), and JiaXian (JX) were used in this study. These varieties were grown under similar planting conditions in an experimental field in Yulin, Shaanxi Province, China in 2021. Purified mature seeds (200 g) were rinsed and turned into flour via a high-speed grinder (FW-100D, XinBaoDe Instruments Ltd., Tianjin, China). Starches were extracted using alkaline steeping methods according to standard procedures of Zhang et al. (27) and Gao et al. (28).

### Physical characteristics of sorghum varieties

Purified seeds were used for the determination of physical characteristics. Random grains were selected from a well-mixed sample and weighed by a digital weighing balance/SC-G Automatic Seed Test Instrument (Wan Shen Testing Company, Hangzhou, China) in three replications to measure the thousand-grain weight, and the mean value was recorded. Sorghum grain color and shape were recorded by visual classification. The grain size distribution, average diameter, and lognormal fitting function of sample grains were measured using the Fiji ImageJ software (ImageJ, US, NIH, Bethesda, MD, USA) and Origin 2018 (OriginLab Corporation, Northampton, MA 01060, USA). Bulk density (BD) was determined using a 10 ml graduated cylinder. A known weight of the sample was poured into the cylinder, and the volume was recorded. BD was calculated using the equation: BD (g/ml) = [mass of the sample (g)/volume of the sample (ml)] (29). The tapped density (TPD) of a sample was calculated using the equation: TPD (g/ml) = [mass of the sample (g)/volume of the sample (ml)] (29). The true density (TD) of the sample was calculated using the formula: TD (g/ml) = mass of the sample (g)/volume of sample displaced by the sand (ml) (29). The percentage of porosity (P) in sample was calculated using the formula: P (%) = (TD–BD)/TD × 100 (30).

### Determination of micrometric properties

The Carr’s index (CI) of the sample was measured as reported by Menaka et al. (29). CI was calculated using the equation: CI = [(TPD–BD)/TPD] × 100. The Hausner’s ratio (HR) was calculated using the formula: HR = (TPD/BD) × 100 (29). To measure the angle of repose (AR), a mounted funnel was placed on a laboratory stand at a height of 10 cm from the bench to measure the AR. About 50 g sample was poured into the mounted funnel with the closed tip. The tip-plug was opened, and the sample flour was allowed to pass through the orifice. The height and diameter of the sample heap were measured. AR was calculated using the equation: AR (θ) = tan^–1^ (h/r), where h represents the height of conical powder heap, and r is the radius of the circular base (29).

### Proximate composition and amylose content analyses of sorghum flours

The moisture, crude fiber, and ash contents were analyzed by standard methods of AOAC (31). Fat (F), protein (PT), and starch contents were determined by Soxhlet extractor, Kjeldahl method, and anthrone spectrophotometry method, respectively (23, 24). The content of carbohydrate (CHO) was calculated by difference: CHO = 100 – (%PT + %F + %moisture + %crude fiber + %ash) (32). The percentage of starch yield (SY) was calculated on dry matter basis of 100 g sample grains. SY was calculated using the following formula: SY (%) = (weight of starch/weight of whole grain) × 100 (30). The starch recovery percent (SR%) of sorghum flour was calculated using the equation: SR (%) = (weight of starch/weight of grain starch) × 100 (32). The amylose (AM) content was analyzed using the method of Yang et al. (33) with some modifications.

### Determination of energy values and percentage of energy contribution

Energy values were determined by multiplying the results of PT, CHO, and F by 17, 17, and 37, respectively (20, 34). Each analysis of the sample was carried out in duplicate. The proportions of energy contributions from F, PT, and CHO to total energy (TE KJ/100 g) were calculated for each proximate composition type. In samples, the utilization of energy due to PT (UEDP%) was also calculated in accordance with the formula of Niyi et al. (35).

### Determination of mineral and amino acid compositions

The analysis of minerals was carried out from the sample solution obtained by dry-ashing the samples at 550°C to constant weight. Sodium and potassium contents were measured using a flame photometer and the phosphorus content was determined by Vanadomolybdate method (20, 36). Other metals (calcium, magnesium, iron, manganese, and zinc) were measured using an atomic absorption spectrophotometer according to the method described by Adeyeye et al. (20). All determinations were measured in triplicate. The AA compositions of flour samples were determined by the pre-column derivatized AccQ.Tag Ultra method and using the reverse-phase HPLC system (37).

### Determination of anti-nutritional factors

The condensed tannin contents of flour samples were determined following the modified vanillin/HCl assay described by Khoddami et al. (38). The absorbance was recorded at 500 nm, and the catechin standard solution was used as template for the condensed tannin assay. Total flavonoids were determined using the assay modified by Khoddami et al. (38) and Afify et al. (39). The reaction mixture was kept for 6 min and then mixed with 2 ml of 1 M NaOH, and the total volume was made up to 10 ml with distilled water. The absorbance and expressed results were 510 nm and μg catechin equivalent/g dry sample (μg/g), respectively (39). Total phenol contents were determined using the Folin-Ciocalteau assay (40, 41) with some modifications. Absorbance was measured at 760 nm. Total phenols were calculated on the basis of standard curves and expressed as mg gallic acid equivalents (GAE)/100 g grain (dry weight, DW).

### Functional properties of sorghum flours

Swelling power (SP) and water solubility (WS) were determined at 90°C by using the methods described by Sindhu and Khatkar (26) and Uarrota et al. (42), respectively. The method of Sindhu and Khatkar (26) was used for the determination of water absorption capacity (WAC) and oil absorption capacity (OAC) of different sorghum flours. Exactly 1.5 g sample suspension was added with 10 ml distilled water, and the mixture was agitated with four times for 10 min. After resting periods of 10 min, sediment samples were centrifuged at 3,250 rpm for 25 min. The supernatant was decanted, and tubes were air dried and then weighed to determine WAC. Approximately 3 ml refined groundnut oil was poured into a known weight of 0.5 g sample, and the mixture was stirred for 1 min and kept for 30 min at room temperature. After 30 min, sample tubes were centrifuged at 3,200 rpm for 25 min. The volume of unabsorbed oil was determined for OAC. Light transmittance (%LT) of flour paste was determined by following the methods of Ghada et al. (19) and Yang et al. (23) with some modifications. The percentage of LT was measured using spectrophotometry (Blue Star B, Lab Tech Ltd., Beijing, China). The methods reported by Sindhu and Khatkar (26) and Thilagavathi et al. (43) were followed with slight modifications for the determination of the least gelation concentration (LGC). Solutions 5 ml of concentration of flour [8–30% (w/v)] in test tubes were placed in a water bath maintained at 90°C and kept overnight at 4°C for cooling. Gelation was recorded by inverting the test tubes for determination of LGC.

### Data analysis

Data were analyzed using the IBM SPSS version 20.0 software (SPSS Inc. Chicago, IL, USA). All data were presented as mean ± standard deviation. Each test was carried out in triplicate. Data were subjected to one-way ANOVA and the Tukey’s multiple range test was performed to compare treatment means. A *p*-value of 0.05 was considered significant. The Pearson correlation was determined using one-way ANOVA on the SPSS version 22.00 and correlation heatmap created on Microsoft Excel 2016. Column bar and Scatterplot matrix graphs were produced using the Origin 2018 software.

## Results and discussion

### Physical characteristics and micrometric properties of sorghum grains

The physical characteristics of sorghum grains were assessed. Results are presented in Table 1. The physical properties of food are important as they are used in product design and development, process optimization, food quality control, and food process modeling (29). Deepa (30) reported that physical properties have unique characteristics a food material responds to physical treatment, including thermal, mechanical, electrical, optical, electromagnetic, and sonic process. The color and shape of all sorghum grains were white and round, respectively. The thousand grain weight had significantly differ (*p* < 0.05). The thousand-grain weights of JZ 34, LZ 19, JN 3, JZ 127, JN 2, and JX significantly differed (*p* < 0.05) and were 22.41, 31.85, 16.23, 35.80, 21.56, and 30.14 g, respectively. JZ 127 had the highest thousand grain weight. The highest values of BD, TPD, and TD were recorded in the LZ 19. In the present study, the TD of LZ 19 was significantly higher (*p* < 0.05) than BD and TPD, which might be due to the filling of void space and pores with sand while determining TD in the sand displacement method (30). Porosity, percentage of air between the particles that compared to a unit volume of particles (44). JZ 34 showed the highest P (44.61%). Deepa (30) stated that the difference in P among various samples is attributed to differences in their density values. Deepa (30) and Devi and Sharma (44) reported that high P resulted in high contact with atmospheric oxygen and high rate of auto-oxidation.

**TABLE 1:** Physical properties of sorghum grains.

Varieties	Grain color	Grain shape	1,000 Grain weight (g)	Bulk density (g/ml)	Tapped density (g/ml)	True density (g/ml)	Porosity (%)
JZ 34	White	Round	22.41 ± 0.71^d^	0.72 ± 0.02^d^	0.88 ± 0.00^b^	1.30 ± 0.01^c^	44.61 ± 1.22^a^
LZ 19	White	Round	31.85 ± 2.25^b^	0.79 ± 0.07^a^	0.96 ± 0.21^a^	1.42 ± 0.11^a^	44.37 ± 0.07^c^
JN 3	White	Round	16.23 ± 0.28^e^	0.75 ± 0.31^b^	0.91 ± 0.12^b^	1.35 ± 0.03^b^	44.44 ± 1.00^b^
JZ 127	White	Round	35.80 ± 0.61^a^	0.74 ± 0.01^c^	0.90 ± 0.10^b^	1.33 ± 0.00^b^	44.36 ± 0.07^c^
JN 2	White	Round	21.56 ± 0.09^d^	0.76 ± 0.02^b^	0.92 ± 0.03^b^	1.36 ± 0.04^b^	44.12 ± 0.00^d^
JX	White	Round	30.14 ± 0.16^c^	0.75 ± 0.12^b^	0.91 ± 0.10^b^	1.35 ± 0.03^b^	44.44 ± 1.00^b^

Data are represented as the mean ± SD of triplicate determinations. For each column, values not displaying the same letter are significantly different (*p* < 0.05). JZ 34, jinza 34; LZ 19, liaoza 19; JN 3, jinnuo 3; JZ 127, jiza 127; JN 2, jiniang 2; JX, jiaxian.

**FIGURE 1:**
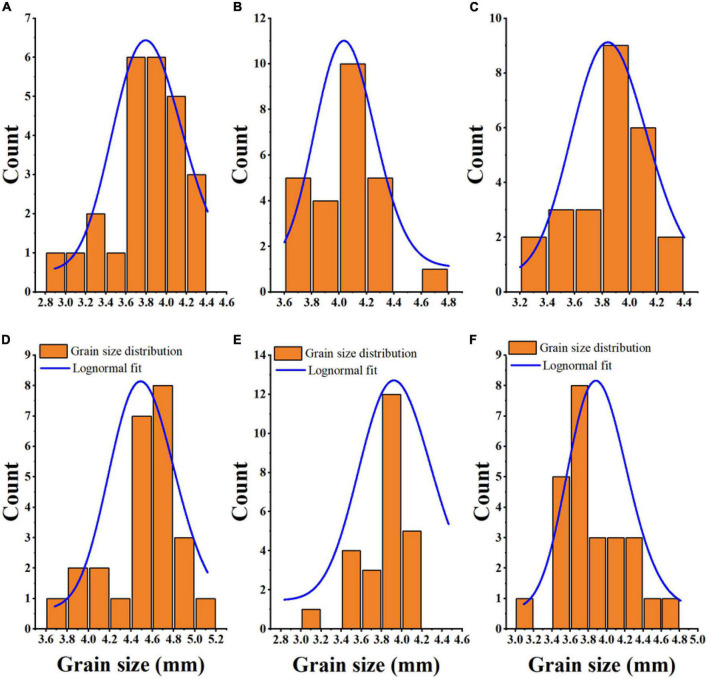
Grain size distribution and Lognormal fitting of six sorghum grains. JZ 34, jinza 34 **(A)**; LZ 19, liaoza 19 **(B)**; JN 3, jinnuo 3 **(C)**; JZ 127, jiza 127 **(D)**; JN 2, jiniang 2 **(E)**; JX, jiaxian **(F)**.

The Fiji ImageJ program is useful as magnetophoretic measurements in observing the physics of particle structure, scientific images (digital and scanning electron microscopy), and scattering-intensity data (9, 45, 46). Figure 1 shows the granule size distribution histogram and lognormal fitting of grain size. According to the Fiji ImageJ and Origin 2018 software results, the average grain sizes of JZ 34, LZ 19, JN 3, JZ 127, JN 2, and JX, were 3.80, 4.05, 3.87, 4.54, 3.83, and 3.87, respectively. The size of sorghum grains ranged from 2.8 to 5.2 mm. The average granule size of JZ 127 (4.54 mm) was larger than those of other varieties. The term micrometric represent to a study of well-derived properties of fine particle in science and technology, it is not only used in the development of pharmaceutical and material science (47), but in widely used in food science and technology for functional, pasting, and formulation (48). The micrometric properties including CI, HR, and AR, of sorghum flours were assessed. Results are shown in Table 2. The above three parameters determined the flow characteristics and compressibility of a powder. In the current study, the CI and AR of all sorghum flours indicated good flow and compressibility properties. Deepa (30) reported that CI (23%) and AR (50°) had good flow and compressibility properties. Our results were consistent into their findings. Among the six sorghum flours, JZ 34 had higher CI and AR values (*p* < 0.05). HR did not significantly differ (*p* > 0.05) in our findings. The good flow properties of granules and powders are important in products designed in compressed form like tablets, and capsules (47), and in the creation of new products, like films and coatings (48).

**TABLE 2:** Micrometric properties of sorghum grains.

Varieties	Carr’s index	Hausner’s ratio	Angle of repose (θ)
JZ 34	18.18 ± 2.10^a^	1.22 ± 0.04^a^	40.25 ± 0.22^a^
LZ 19	17.71 ± 1.20^c^	1.21 ± 0.00^a^	39.92 ± 0.20^b^
JN 3	17.58 ± 1.11^d^	1.21 ± 0.00^a^	39.92 ± 0.21^b^
JZ 127	17.78 ± 1.22^b^	1.22 ± 0.04^a^	40.25 ± 0.22^a^
JN 2	17.39 ± 1.00^e^	1.21 ± 0.01^a^	39.92 ± 0.20^b^
JX	17.58 ± 1.11^d^	1.21 ± 0.03^a^	39.92 ± 0.20^b^

Data are represented as the mean ± SD of triplicate determinations. For each column, values not displaying the same letter are significantly different (*p* < 0.05). JZ 34, jinza 34; LZ 19, liaoza 19; JN 3, jinnuo 3; JZ 127, jiza 127; JN 2, jiniang 2; JX, jiaxian.

### Proximate compositions and energy values of sorghum varieties

The proximate composition was significantly influenced by six sorghum varieties (Table 3). The highest values of crude fiber (2.49%), ash (1.91%), PT (9.34%), and F (5.14%) were recorded in JN 3. In this study, LZ 19 had the lowest crude fiber (1.58%) and F (2.98%) contents, whereas JX showed the lowest PT content (6.24%). Table 3 shows that JX contained considerably CHO content (78.46%), starch content (77.42%), SY (69.43%), and starch recovery (89.68%) than other sorghum varieties. In summary, our results showed that among sorghum varieties, JN 3 and JX had nutritional potential in terms of proximate composition.

**TABLE 3:** Proximate compositions of sorghum grains.

Proximate compositions (%)	JZ 34	LZ 19	JN 3	JZ 127	JN 2	JX
Moisture	9.53 ± 0.18^b^	9.66 ± 0.14^a^	9.49 ± 0.24^b^	8.30 ± 0.19^d^	8.89 ± 0.21^c^	8.04 ± 0.24^e^
Crude fiber	2.13 ± 0.05^b^	1.58 ± 0.05^d^	2.49 ± 0.05^a^	2.20 ± 0.19^b^	2.04 ± 0.11^b^	1.80 ± 0.09^c^
Ash	1.53 ± 0.07^c^	1.31 ± 0.02^e^	1.91 ± 0.12^a^	1.30 ± 0.03^e^	1.45 ± 0.06^d^	1.75 ± 0.08^b^
Protein	8.31 ± 0.03^c^	8.91 ± 0.11^b^	9.34 ± 0.25^a^	8.84 ± 0.30^b^	8.38 ± 0.20^c^	6.24 ± 0.03^d^
Fat	3.49 ± 0.32^b^	2.98 ± 0.19^c^	5.14 ± 0.26^a^	3.67 ± 0.40^b^	4.02 ± 0.46^b^	3.71 ± 0.26^b^
Carbohydrate	75.01 ± 0.10^c^	75.56 ± 0.11^b^	71.63 ± 0.09^d^	75.69 ± 0.14^b^	75.22 ± 0.21^b^	78.46 ± 0.35^a^
Starch	73.82 ± 0.29^b^	75.24 ± 0.42^b^	70.26 ± 0.23^c^	74.98 ± 0.37^b^	74.54 ± 0.19^b^	77.42 ± 0.50^a^
Starch yield	65.83 ± 0.13^c^	67.25 ± 0.21^b^	62.27 ± 0.05^d^	66.99 ± 0.41^b^	66.55 ± 0.11^c^	69.43 ± 0.71^a^
Starch recovery	89.18 ± 0.04^c^	89.38 ± 1.30^b^	88.63 ± 0.02^c^	89.34 ± 1.10^b^	89.28 ± 0.50^b^	89.68 ± 0.08^a^

Data are represented as the mean ± SD of triplicate determinations. Along the same row, values having different letter vary significantly different (*p* < 0.05). JZ 34, jinza 34; LZ 19, liaoza 19; JN 3, jinnuo 3; JZ 127, jiza 127; JN 2, jiniang 2; JX, jiaxian.

Table 4 depicts the percentage of energy values contributed by PT (PEP%), F (PEF%), CHO (PEC%), and utilizable energy due to PT (UEDP%). PEP, PEF, PEC, and UEDP in sorghum flours were 9.07–10.13%, 7.13–12.14%, 77.73–84.57%, and 4.04–6.08%, respectively. The JN 3 with the highest concentrations of F and PT also had the highest proportions of energy contributions due to F (PEF, 12.14%), PT (PEP, 10.13%), and UEDP (6.08%). The energy contribution by CHO was highest in the JX sorghum sample (84.57%). The total energy (TE) values of JZ 34, LZ 19, JN 3, JZ 127, JN 2, and JX were 1545.57, 1546.25, 1577.80, 1566.67, 1569.94, and 1572.17 KJ, respectively. These results indicated that JN 3 and JX had the highest TE values than others. Adeyeye et al. (20) reported that proximate compositions, and energy values and contributions in raw sorghum were higher than those in steeped and germinated sorghum samples.

**TABLE 4:** Energy values of different sorghum grains.

Varieties	Total energy	PEP	PEF	PEC	UEDP
	(KJ)	(%)	(%)	(%)	(%)
JZ 34	1545.57 ± 2.20^d^	9.14 ± 0.02^c^	8.35 ± 0.02^c^	82.50 ± 0.04^c^	5.48 ± 0.12^c^
LZ 19	1546.25 ± 1.01^d^	9.80 ± 0.09^b^	7.13 ± 0.03^d^	83.07 ± 0.02^b^	5.88 ± 0.10^b^
JN 3	1577.80 ± 0.03^a^	10.13 ± 1.00^a^	12.14 ± 0.14^a^	77.73 ± 0.01^d^	6.10 ± 0.06^a^
JZ 127	1566.67 ± 0.09^c^	9.55 ± 0.03^b^	8.63 ± 0.11^c^	81.81 ± 0.06^c^	5.73 ± 0.08^b^
JN 2	1569.94 ± 0.0.11^c^	9.07 ± 0.06^c^	9.47 ± 0.09^b^	81.45 ± 0.04^c^	5.44 ± 0.06^c^
JX	1572.17 ± 9.02^b^	6.73 ± 0.00^d^	8.70 ± 0.04^c^	84.57 ± 0.11^a^	4.04 ± 0.03^d^

Data are represented as the mean ± SD of triplicate determinations. For each column, values not displaying the same letter are significantly different (*p* < 0.05). JZ 34, jinza 34; LZ 19, liaoza 19; JN 3, jinnuo 3; JZ 127, jiza 127; JN 2, jiniang 2; JX, jiaxian. PEP, proportion of total energy due to protein; PEF, proportion of total energy due to fat; PEC, proportion of total energy due to carbohydrate; UEDP, utilizable energy due to protein.

### Scatterplot matrix analysis of some proximate parameters and energy values

**FIGURE 2:**
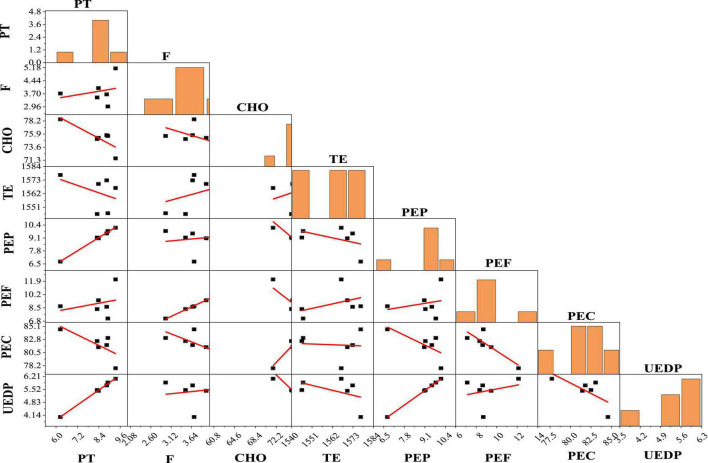
Scatterplot matrix analysis of protein (PT), fat (F), carbohydrate (CHO), and energy values contributions. TE, total energy; PEP, proportion of total energy due to protein; PEF, proportion of total energy due to fat; PEC, proportion of total energy due to carbohydrate; UEDP, utilizable energy due to protein.

The scatterplot matrices of PT, F, CHO, and energy values and its contributions (TE, PEP, PEF, PEC, and UEDP) are shown in Figure 2. PEP, PEF, and PEC were highly positively correlated with PT (*r* = 0.998), F (*r* = 0.999), and CHO (*r* = 0.952, *p* < 0.05), respectively. Based on scatterplot analysis, TE had a positive correlation with PEF (*r* = 0.428) and negative correlation with PEP (*r* = -0.456) and PEC (*r* = -0.073), respectively. Furthermore, UEDP had a strongly significant positive correlation with PEP (*r* = 1.000, *p* < 0.05).

### Amylose analysis

**FIGURE 3:**
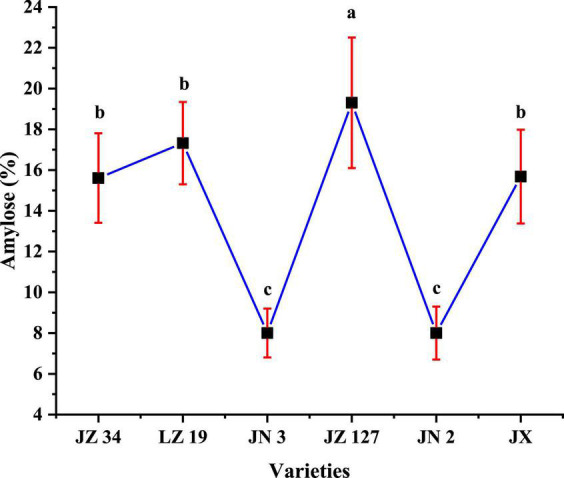
Amylose content of sorghum flours. JZ 34, jinza 34; LZ 19, liaoza 19; JN 3, jinnuo 3; JZ 127, jiza 127; JN 2, jiniang 2; JX, jiaxian. The data showed the mean of three replications, and error bars are standard deviations. Different letters **(a–c)** indicate there were significant differences (*p* < 0.05) in the LSD mean comparisons between the treatments mean.

The amylose content affects the physicochemical and functional properties of flour and starch (22, 26). The amylose contents of JZ 34, LZ 19, JN 3, JZ 127, JN 2, and JX were 15.61, 17.32, 8.00, 19.30, 8.00, and 15.68%, respectively (Figure 3). In our studies, JZ 127 had the highest amylose content (19.30%), whereas JN 3 (8.00%) and JN 2 (8.00%) had the lowest amylose content.

### Minerals and anti-nutritional quality traits of sorghum

In the present study, the different sorghum flours significantly affected the mineral contents (Table 5). However, the highest values of calcium (0.04%), phosphorus (0.35%), potassium (0.38%), sodium (0.05%), magnesium (0.19%), iron (50.00%), manganese (16.30%), and zinc (15.40%) were found in JN 3. Sorghum grains have higher mineral contents than other cereals, including rice (49), wheat (50), millet (51), and maize (52).

**TABLE 5:** Mineral compositions of different sorghum grains.

Mineral compositions (%)	JZ 34	LZ 19	JN 3	JZ 127	JN 2	JX
Calcium	0.02 ± 0.01^b^	0.01 ± 0.00^b^	0.04 ± 0.01^a^	0.02 ± 0.01^b^	0.01 ± 0.00^b^	0.02 ± 0.01^b^
Phosphorus	0.20 ± 0.01^c^	0.21 ± 0.01^c^	0.35 ± 0.03^a^	0.23 ± 0.02^b^	0.17 ± 0.03^d^	0.16 ± 0.00^d^
Potassium	0.15 ± 0.01^d^	0.22 ± 0.02^b^	0.38 ± 0.02^a^	0.14 ± 0.00^d^	0.23 ± 0.02^b^	0.17 ± 0.03^c^
Sodium	0.01 ± 0.00^c^	0.03 ± 0.00^b^	0.05 ± 0.00^a^	0.02 ± 0.00^b^	0.02 ± 0.00^b^	0.01 ± 0.00^c^
Magnesium	0.12 ± 0.01^c^	0.09 ± 0.00^d^	0.19 ± 0.02^a^	0.11 ± 0.01^c^	0.13 ± 0.02^b^	0.11 ± 0.01^c^
Iron	37.00 ± 0.21^b^	38.00 ± 0.20^b^	50.00 ± 0.33^a^	36.00 ± 0.20^b^	32.00 ± 0.11^c^	33.00 ± 0.12^c^
Manganese	13.20 ± 0.01^e^	14.00 ± 0.05^d^	16.30 ± 0.06^a^	14.21 ± 0.06^c^	14.00 ± 0.00^d^	14.50 ± 0.03^b^
Zinc	14.21 ± 0.06^d^	14.90 ± 0.04^b^	15.40 ± 0.07^a^	14.30 ± 0.02^c^	14.11 ± 0.02^e^	14.20 ± 0.06^d^

Data are represented as the mean ± SD of triplicate determinations. Along the same row, values having different letter vary significantly different (*p* < 0.05). JZ 34, jinza 34; LZ 19, liaoza 19; JN 3, jinnuo 3; JZ 127, jiza 127; JN 2, jiniang 2; JX, jiaxian.

**FIGURE 4:**
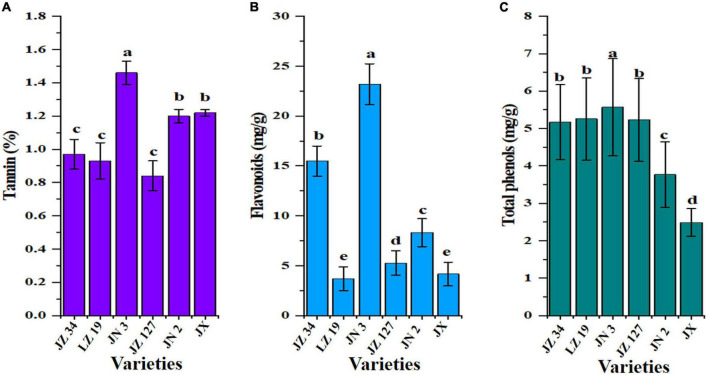
Anti-nutritional factors of six sorghum grains, tannin **(A)**, flavonoids **(B)**, and total phenols **(C)**. JZ 34, jinza 34; LZ 19, liaoza 19; JN 3, jinnuo 3; JZ 127, jiza 127; JN 2, jiniang 2; JX, jiaxian. The data showed the mean of three replications, and error bars are standard deviations. Different letters **(a–e)** indicate there were significant differences (*p* < 0.05) in the LSD mean comparisons between the treatments mean.

Anti-nutritional contents, such as tannin, flavonoids, and total phenols, are shown in Figure 4. Sorghum consists of two main anti-nutritional factors, namely, tannin and total phenols, which are located in the grain (53). The major anti-nutritional effects of tannins are reduction in feed intake, thus diminishing the digestibility and utilization of nutrients and adversely affecting the metabolism and toxicity in the livestock industry (53). Khoddami et al. (38) and Shen et al. (41) reported that the level of tannins present in sorghum can be the dominant factor that influences its nutritional value for food and non-food industries. In our studies, JN 3 flour had the highest tannin (1.46%), flavonoid (23.19 mg/g), and total phenol (5.57 mg/g) contents than other sorghum flours (Figures 4A–C). Tannin contents ranged from 0.01 to 2.12% (54) who studied 110 Chinese sorghum grains for determination of tannin contents by near-infrared reflectance spectroscopy (NIRS) and designated as tannin-free. The grains of all sorghums were observed free of tannin. Awika (55) stated that sorghums lacking a pigment testa are considered “free of tannin.” Different grain colors in the same species and growing environment (41, 56), result in differences in the total phenol contents of varieties.

### Amino acid composition analysis

The AA contents of different sorghum varieties are summarized in Table 6. JN 3 had the highest essential contents of essential (Thr, Val, Met, Ile, Leu, and Phe), basic (Arg, His, and Lys), acidic (Asp), hydrophobic (Ala), and polar uncharged (Gly and Tyr) AAs. A high Arg content can be used for the treatment of cardiovascular diseases (57, 58). Lys is the first limiting AA in cereal grains, which are staple food (57). Li et al. (21) reported that Glu and Asp are the primary AAs of seed storage PT, and are acidic AA. A high concentration of hydrophobic AA (Ala) affected the functional properties of PTs and provided the dense internal structure of PT form, thus improving its thermal stability (59, 60). In summary, our result showed that JN 3 sorghum grain had remarkable potential for nutritional supplement in industrial food applications.

**TABLE 6:** Amino acids compositions of different sorghum grains.

Parameters (%)	JZ 34	LZ 19	JN 3	JZ 127	JN 2	JX
Thr	0.03 ± 0.00^c^	0.06 ± 0.00^b^	0.08 ± 0.00^a^	0.04 ± 0.00^c^	0.06 ± 0.00^b^	0.06 ± 0.00^b^
Val	0.37 ± 0.01^d^	0.69 ± 0.02^c^	0.90 ± 0.03^a^	0.34 ± 0.01^e^	0.80 ± 0.03^b^	0.71 ± 0.02^c^
Met	0.05 ± 0.00^b^	0.05 ± 0.00^b^	0.07 ± 0.00^a^	0.04 ± 0.00^b^	0.05 ± 0.00^b^	0.05 ± 0.00^b^
Ile	0.27 ± 0.01^d^	0.52 ± 0.02^c^	0.67 ± 0.02^a^	0.24 ± 0.01^e^	0.60 ± 0.02^b^	0.53 ± 0.01^c^
Leu	0.76 ± 0.02^e^	1.86 ± 0.08^c^	2.32 ± 0.07^a^	0.71 ± 0.02^f^	2.21 ± 0.20^b^	1.71 ± 0.02^d^
Phe	0.17 ± 0.01^d^	0.37 ± 0.01^b^	0.41 ± 0.01^a^	0.15 ± 0.01^e^	0.36 ± 0.01^b^	0.33 ± 0.01^c^
His	0.03 ± 0.00^c^	0.05 ± 0.00^b^	0.07 ± 0.00^a^	0.02 ± 0.00^d^	0.04 ± 0.00^c^	0.05 ± 0.00^b^
Lys	0.03 ± 0.01^c^	0.02 ± 0.00^c^	0.31 ± 0.00^a^	0.03 ± 0.00^c^	0.02 ± 0.00^c^	0.18 ± 0.01^b^
Asp	0.11 ± 0.01^e^	0.41 ± 0.01^d^	0.61 ± 0.02^a^	0.54 ± 0.01^b^	0.51 ± 0.01^c^	0.52 ± 0.01^c^
Gly	0.01 ± 0.00^b^	0.01 ± 0.00^b^	0.03 ± 0.00^a^	0.01 ± 0.00^b^	0.01 ± 0.00^b^	0.01 ± 0.00^b^
Ala	0.63 ± 0.02^c^	1.65 ± 0.06^b^	2.21 ± 0.20^a^	0.60 ± 0.02^d^	2.00 ± 0.03^b^	1.67 ± 0.02^b^
Tyr	0.03 ± 0.00^c^	0.06 ± 0.00^b^	0.08 ± 0.00^a^	0.01 ± 0.00^d^	0.04 ± 0.00^c^	0.06 ± 0.00^b^
Arg	0.09 ± 0.00^d^	0.13 ± 0.01^c^	0.20 ± 0.01^a^	0.04 ± 0.00^e^	0.17 ± 0.01^b^	0.10 ± 0.01^d^

Data are represented as the mean ± SD of triplicate determinations. Along the same row, values having different letter vary significantly different (*p* < 0.05). JZ 34, jinza 34; LZ 19, liaoza 19; JN 3, jinnuo 3; JZ 127, jiza 127; JN 2, Jiniang 2; JX, jiaxian. Thr, thereonine; Val, valine; Met, methionine; Ile, isoleucine; Leu, leucine; Phe, phenaylalanine; His, histidine; Lys, lysine; Asp, aspartic acid; Gly, glycine; Ala, alanine; Tyr, tyrosine; Arg, arginine.

### Functional properties

**FIGURE 5:**
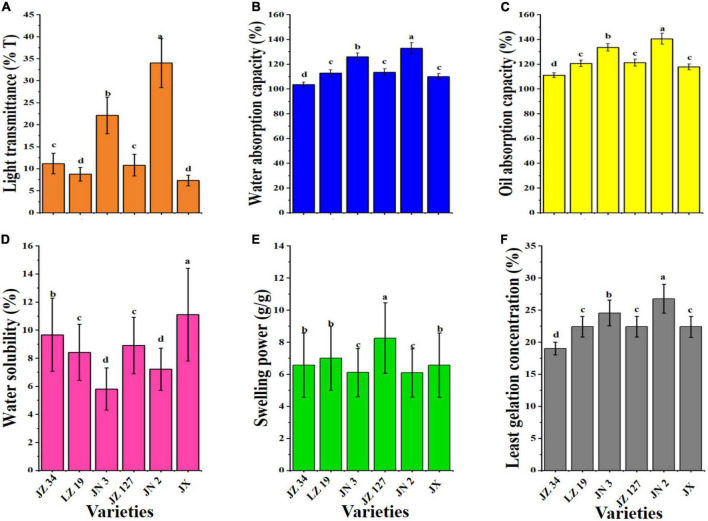
Functional properties of sorghum grains. Light transmittance **(A)**, water absorption capacity **(B)**, oil absorption capacity **(C)**, water solubility **(D)**, swelling power **(E)**, and least gelation concentration **(F)**. JZ 34, jinza 34; LZ 19, liaoza 19; JN 3, jinnuo 3; JZ 127, jiza 127; JN 2, jiniang 2; JX, jiaxian. The data showed the mean of three replications, and error bars are standard deviations. Different letters **(a–d)** indicate there were significant differences (*p* < 0.05) in the LSD mean comparisons between the treatments mean.

Among other sorghum grains, JN 2 had the highest LT (34.01%, Figure 5A). Sindhu and Khatkar (26) reported that the AM content affects the transmittance value of paste, which can be responsible for the difference in the turbidity of sorghum flour in the current study. JN 2 had the highest LT (34.01%) and decreased AM content, and these findings were consistent with some studies pointing out that an increase in AM content will decreases the transparency of flour paste (61). The WAC is the ability of the flour to maintain water against gravity and is improved by PT and CHO by supporting hydrophilic parameters, like polar and charged side chains (26). The WAC of different sorghum varieties ranged from 103.43 to 132.86%, and among the samples, JN 2 had the highest WAC (132.86%, Figure 5B). In the food industry, the role of OAC is the interaction between the non-polar AA side chains and hydrocarbon chains of lipid to determine mouthfeel and flavor retention of products (62). As shown in Figure 5C, JN 2 had high OAC (140.52%), whereas JZ 34 had the lowest OAC (111.09%).

The functions of SP and WS are the disruption and breakage of hydrogen bonds between water molecules and AM and amylopectin (63). JX had the highest WS (11.11%), and JN 2 had the lowest WS (7.22%, Figure 5D). Figure 5E shows that JZ 127 had the highest SP (8.25 g/g) compared with other treatments. In our study, among sorghum varieties, SP and WS were different, which might be due to the AM content (9, 26). Uarrota et al. (42) and Yang et al. (23) reported that AM inhibits the SP and WS in the physicochemical properties of cereal starch. The LGC is the amount of starch and gelation of pasting properties (26). The LGC of JZ 34, LZ 19, JN 3, JZ 127, JN 2, and JX were 19.00, 22.42, 24.55, 22.40, 26.77, and 22.39%, respectively (Figure 5F). In our studies, JN 2 had the highest LGC (26.77%), whereas JZ 34 had the lowest LGC (19.00%). LGC could be added as composite food for curd formation and could be used as additives of food materials for forming gel in food products (64). The low LGC of flour is required for improved gelling formation of PT ingredients, and resulting in increased SP of the flour (65, 66).

### Correlation heatmap analysis

**FIGURE 6:**
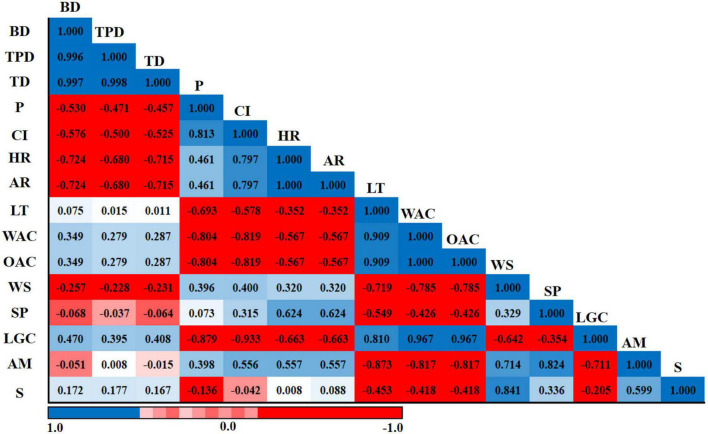
Pearson’s correlation coefficients of amylose, starch, physical properties, micrometric properties, and functional properties of six sorghum grains. BD, bulk density; TPD, tapped density; TD, true density; P, porosity; CI, Carr’s index; HR, Hausner’s ratio; AR, angle of repose; LT, light transmittance; WAC, water absorption capacity; OAC, oil absorption capacity; WS, water solubility; SP, swelling power; LGC, least gelation concentration; AM, amylose; S, starch. The numbers in each field represent the correlation extent; the color represents significant correlation (*p* < 0.05); the deeper the color of the field, the more significant the correlation (*p* < 0.01). The blue color means a positive correlation, and the red color means a negative correlation.

The Pearson correlation results of amylose, starch, physical properties, micrometric properties, and functional properties of six sorghum grains are presented in Figure 6. BD was positively correlated with TPD (*r* = 0.996) and TD (*r* = 0.997). TD had a strong positive correlation with TPD (*r* = 0.998), whereas CI had highly positive association with P (*r* = 0.813). AR had a strongly significant positive correlation with HR (*r* = 1.000). WAC had a highly positive correlation with LT (*r* = 0.909). By contrast, WAC was negatively correlated with CI (*r* = -0.819). OAC had a highly negative correlation with CI (*r* = -0.819) and a highly positive association with LT (*r* = 0.909). Furthermore, OAC had a strongly significant positive correlation with WAC (*r* = 1.000). LGC was strongly and negatively correlated with CI (*r* = -0.933). Moreover, LGC was strongly and positively correlated with WAC (*r* = 0.967) and OAC (*r* = 0.967). AM showed a highly negative association with LT (*r* = -0.873), WAC (*r* = -0.817), and OAC (*r* = -0.817). In addition, AM had a highly positive relationship with WS (*r* = 0.714) and SP (*r* = 0.824).

## Conclusion

We studied the physical properties, nutritive quality, and functional properties of six different sorghum varieties. The flour of JN 127 had the highest amylose content. The physical properties of LZ 19 were better than those of other sorghum grains, and JZ 34 had the best micrometric properties. JX produced higher SY and was suitable as a frozen food thickener or food additive and raw material for porridge, couscous, and mayonnaise. All sorghums were free of tannin. Among the six sorghum grains, JN 3 had the highest proximate, mineral, and AA compositions and energy contributions. The six sorghum grains had differences in functional properties, including LT, WAC, OAC, WS, SP, and LGC of flours as composite flours products in sorghum for healthy food. Our results indicated that JN 3 could be utilized for brewing sorghum grain and liquor flavor and could be effective materials for functional foods to improve health. Our assessments in future will stimulate the utilization of sorghum as a potential crop in dryland sustainable agro-ecosystems and rural livelihood nutritional security in arid and semiarid regions of northern China.

## Data availability statement

The original contributions presented in this study are included in the article/supplementary material, further inquiries can be directed to the corresponding author.

## Author contributions

MH: conceptualization, investigation, methodology, data, software, writing—original draft preparation, and writing—review and editing. BF: writing—review and editing, supervision, funding acquisition, project administration, and resources. HW and LT: investigation and methodology. VY: writing—review and editing. All authors have read and approved the final version of the manuscript.
